# Supplementary material for: Control of entropy in neural models of environmental state
Source: eLife. 2019 Feb 28;8:e39404. doi: 10.7554/eLife.39404 (PMC6395063; doi:10.7554/eLife.39404)
Supplement: Transparent reporting form [file elife-39404-transrepform.docx]

***eLife’s* transparent reporting form**

We encourage authors to provide detailed information *within their submission* to facilitate the interpretation and replication of experiments. Authors can upload supporting documentation to indicate the use of appropriate reporting guidelines for health-related research (see [EQUATOR Network](http://www.equator-network.org/%20)), life science research (see the [BioSharing Information Resource](https://biosharing.org/)), or the [ARRIVE guidelines](http://www.plosbiology.org/article/info:doi/10.1371/journal.pbio.1000412) for reporting work involving animal research. Where applicable, authors should refer to any relevant reporting standards documents in this form.

If you have any questions, please consult our Journal Policies and/or contact us: [editorial@elifesciences.org](mailto:editorial@elifesciences.org).

**Sample-size estimation**

- You should state whether an appropriate sample size was computed when the study was being designed
- You should state the statistical method of sample size computation and any required assumptions
- If no explicit power analysis was used, you should describe how you decided what sample (replicate) size (number) to use

Please outline where this information can be found within the submission (e.g., sections or figure legends), or explain why this information doesn’t apply to your submission:

This information can be found in the “Participants” section of the “Methods” section.

**Replicates**

- You should report how often each experiment was performed
- You should include a definition of biological versus technical replication
- The data obtained should be provided and sufficient information should be provided to indicate the number of independent biological and/or technical replicates
- If you encountered any outliers, you should describe how these were handled
- Criteria for exclusion/inclusion of data should be clearly stated
- High-throughput sequence data should be uploaded before submission, with a private link for reviewers provided (these are available from both GEO and ArrayExpress)

Please outline where this information can be found within the submission (e.g., sections or figure legends), or explain why this information doesn’t apply to your submission:

We did not encounter or exclude outliers. The only data that were excluded were entire datasets due to technical difficulties that affected data quality, as stated in the “Participants” section.

There was no independent replication of the experiment. However in some cases to check robustness, we tested that analyses give similar results when the exact analysis method used is varied. One important result on which the later analyses are premised, reported in Figure 2b, where we describe a regression of choice decodability on model entropy. We show in the Figure 2 supplements that the same brain region is found if model entropy is replaced with related regressors namely probability assigned to the currently selected option, and the difference between high and low exploit periods.

**Statistical reporting**

- Statistical analysis methods should be described and justified
- Raw data should be presented in figures whenever informative to do so (typically when N per group is less than 10)
- For each experiment, you should identify the statistical tests used, exact values of N, definitions of center, methods of multiple test correction, and dispersion and precision measures (e.g., mean, median, SD, SEM, confidence intervals; and, for the major substantive results, a measure of effect size (e.g., Pearson's r, Cohen's d)
- Report exact p-values wherever possible alongside the summary statistics and 95% confidence intervals. These should be reported for all key questions and not only when the p-value is less than 0.05.

Please outline where this information can be found within the submission (e.g., sections or figure legends), or explain why this information doesn’t apply to your submission:

There are numerous statistical tests reported in the manuscript. In each case we have reported using the following format:

t-tests:

“There was a difference between X and Y (t(n)=t-score, p=p-value)”. Note that for all t-tests, in accordance with Normal assumptions, the comparison is difference of means, the measure of spread is standard deviation, and N is given by the degrees of freedom +1, e.g. t(18) means N=19. We have not stated these assumptions explicitly for each t-test, as these are the only correct options for Student’s t-test.

Linear regression analyses:

In each case we report the co-regressors used in the general linear model in the section “fMRI analyses” in “Methods” (all GLM analyses are numbered). The statistical significance of regressions across the group was evaluated using t-tests, reported as above.

Multiple comparisons correction:

In whole brain image analysis, we used multiple comparisons correction based on cluster mass, this is described in “fMRI analyses” of the “Methods” section.

(For large datasets, or papers with a very large number of statistical tests, you may upload a single table file with tests, Ns, etc., with reference to sections in the manuscript.)

**Group allocation**

- Indicate how samples were allocated into experimental groups (in the case of clinical studies, please specify allocation to treatment method); if randomization was used, please also state if restricted randomization was applied
- Indicate if masking was used during group allocation, data collection and/or data analysis

Please outline where this information can be found within the submission (e.g., sections or figure legends), or explain why this information doesn’t apply to your submission:

This was a within-subjects design as is typical for functional MRI studies. Therefore participants were not allocated to groups.

**Additional data files (“source data”)**

- We encourage you to upload relevant additional data files, such as numerical data that are represented as a graph in a figure, or as a summary table
- Where provided, these should be in the most useful format, and they can be uploaded as “Source data” files linked to a main figure or table
- Include model definition files including the full list of parameters used
- Include code used for data analysis (e.g., R, MatLab)
- Avoid stating that data files are “available upon request”

Please indicate the figures or tables for which source data files have been provided:

1. We will upload, on acceptance, the brain maps to NeuroVault (URL: https://neurovault.org/collections/4872/).

2. We have uploaded the MATLAB code for the Bayesian model in Figure 1, and the behavioural datasets to which the model was fit. No additional parameters are needed to run this model, all non-free parameters are hard coded and also described in the Supplementary section “Bayesian learning model”. The code and data have been uploaded as a source file.

3. We have uploaded the epoched pupil data to Dryad (URL: https://datadryad.org/review?doi=doi:10.5061/dryad.jk17vk0), which together with the behavioural data (point 2 above) can be used to replicate pupil analyses.

4. Individual participant data cannot be publicly shared as the ethical approval for our study does not permit this. Due to their large size (>18GB), it’s not practical to upload the multivariate fMRI analyses, but these are available on request from the corresponding author.

6. However, for the multivariate analyses, we are able to, and have, uploaded the medial OFC ROI data as a source data file (Figure 2-source data 1). This can be used to replicate the key multivariate results.
